# Ecotoxicological Assessment of Thermally- and Hydrogen-Reduced Graphene Oxide/TiO_2_ Photocatalytic Nanocomposites Using the Zebrafish Embryo Model

**DOI:** 10.3390/nano9040488

**Published:** 2019-03-28

**Authors:** Halema Al-Kandari, Nadin Younes, Ola Al-Jamal, Zain Z. Zakaria, Huda Najjar, Farah Alserr, Gianfranco Pintus, Maha A. Al-Asmakh, Aboubakr M. Abdullah, Gheyath K. Nasrallah

**Affiliations:** 1 Department of Health Environment, College of Health Sciences, PAAET, P.O. Box 1428, Faiha, 72853 Kuwait City, Kuwait; ha1.alkandari@paaet.edu.kw; 2 Department of Biomedical Science, College of Health Sciences, QU Health, Qatar University, Doha 2713, Qatar; ny1204022@qu.edu.qa (N.Y.); hn1517144@qu.edu.qa (H.N.); fa1513310@qu.edu.qa (F.A.); gpintus@qu.edu.qa (G.P.); maha.alasmakh@qu.edu.qa (M.A.A.-A.); 3 Biomedical Research Center, QU Health, Qatar University, Doha 2713, Qatar; ola.aljamal@qu.edu.qa (O.A.-J.); zz1513224@qu.edu.qa (Z.Z.Z.); 4 Department of Chemical Engineering, College of Engineering, Doha, Qatar University, Doha 2713, Qatar; 5 Center for Advanced Materials, Qatar University, Doha 2713, Qatar

**Keywords:** reduced graphene oxide/TiO_2_, nanocomposite photocatalysts, zebrafish, toxicity, LC_50_

## Abstract

Advanced oxidation processes (AOPs) have recently attracted great interest in water pollution management. Using the zebrafish embryo model, we investigated the environmental impacts of two thermally (RGOTi)- and hydrogen (H_2_RGOTi)-reduced graphene oxide/TiO_2_ semiconductor photocatalysts recently employed in AOPs. For this purpose, acutoxicity, cardiotoxicity, neurobehavioral toxicity, hematopoietic toxicity, and hatching rate were determinate. For the RGOTi, the no observed effect concentration (NOEC, mortality/teratogenicity score <20%) and the median lethal concentration (LC_50_) were <400 and 748.6 mg/L, respectively. H_2_RGOTi showed a NOEC similar to RGOTi. However, no significant mortality was detected at all concentrations used in the acutoxicity assay (up to1000 mg/L), thus indicating a hypothetical LC_50_ higher than 1000 mg/L. According to the Fish and Wildlife Service Acute Toxicity Rating Scale, RGOTi can be classified as “practically not toxic” and H_2_RGOTi as “relatively harmless”. However, both nanocomposites should be used with caution at concentration higher than the NOEC (400 mg/L), in particular RGOTi, which significantly (i) caused pericardial and yolk sac edema; (ii) decreased the hatching rate, locomotion, and hematopoietic activities; and (iii) affected the heart rate. Indeed, the aforementioned teratogenic phenotypes were less devastating in H_2_RGOTi-treated embryos, suggesting that the hydrogen-reduced graphene oxide/TiO_2_ photocatalysts may be more ecofriendly than the thermally-reduced ones.

## 1. Introduction

According to the United States Environmental Protection Agency (US-EPA), phenolic compounds such as phenol, p-nitrophenol (NP) and p-chlorophenol (CP) are common pollutants with harmful effects on both humans and the ecosystem [1,2,3,4,5,6,7]. To overcome this problem, new techniques are being tested to treat both phenolic compound- and dye-contaminated water. For example, the advanced oxidation processes (AOPs), which is a set of oxidative chemical treatment procedures aimed to remove organic/inorganic materials in water/wastewater [8], use semiconductor photocatalysts such as TiO_2_, ZnO, CdS, ZnS, and GaP to decompose a diversity of refractory pollutants [9,10,11,12]. Besides its promising chemical and biological stability, TiO_2_ is also an accessible and tailorable semiconductor photocatalyst [3,13,14,15,16]. Nevertheless, having a high electron-hole recombination rate and wide bandgap energy, TiO_2_ cannot be excited under visible light irradiation (λ > 400 nm) [17,18]. To gain insight into this specific issue, intensive research work has been directed towards the development of visible light-responsive TiO_2_ with high catalytic activity using co-catalysts such as carbon nitride or reduced graphene oxide (RGO) because of their exceptional electrical properties and controllable structures [17,19,20,21,22,23,24,25].

In this regard, we successfully prepared and studied the photocatalytic activity of 0.1% RGO/TiO_2_ under visible light illumination for phenol degradation [17]. The degradation rate reached 84%, 93%, and 99% within 30 min for phenol, p-chlorophenol and p-nitrophenol respectively [17]. We also demonstrated that different RGO preparation methods might affect the RGO/TiO_2_ photocatalytic activity [12,26]. For example, Thermally-reduced graphene oxide/TiO_2_ (RGOTi) and hydrogen-reduced graphene oxide/TiO_2_ (H_2_RGOTi) photocatalysts have different photocatalytic activities due to their band gap energy (2.96 and 3.03 eV for RGOTi and H_2_RGOTi, respectively) difference. Although our nanocomposite photocatalysts (RGOTi and H_2_RGOTi) have shown excellent performance in both degradation and mineralization of phenolic refractory nanocomposites, they have never been systematically evaluated for safety or any other potential harmful impact on the environment with particular respect to the aquatic fauna and/or flora.

Given that our newly synthesized photocatalytic nanocomposites will be essentially employed in water, this study has been undertaken to evaluate their potential toxic effects on the zebrafish (*Danio rerio*) embryo as a model of aquatic fauna toxicity. In order to find the no observed effect concentration (NOEC), the lowest observed effect concentration (LOEC), and the median lethal concentration (LC_50_), we tested a wide range of concentrations (100, 200, 300, 400, 600, 800, 1000 mg/L). Then, using different teratogenic toxicity assays (hatching rate, cardiotoxicity, locomotion and hematopoietic activities), we investigated the potential adverse effects of the nanocomposite’s high concentration on the normal development of zebrafish at the early stages of life.

## 2. Materials and Methods

### 2.1. Chemicals

Zinc oxide (ZnO) nanopowder, <100 nm particle size was obtained from Sigma-Aldrich (St. Louis, MO, USA). This nanoparticle was used as positive control (PC) in our acutoxicity assays because it is known to cause mortality and teratogenic effects in zebrafish embryos [27,28,29]. N-phenylthiourea (PTU) (Sigma, Steinheim, Germany) in egg water (E3 media) was used as a media to raise zebrafish embryos in vitro. In addition, it is used to inhibit pigment formation in the developing zebrafish embryos to facilitate their visualization under the microscope. E3 media (used to cultivate zebrafish embryos) constituents including 5.0 mM sodium chloride (NaCl), 0.17 mM potassium chloride (KCl), 0.33 mM magnesium sulfate heptahydrate (MgSO_4_·7H_2_O) and 0.33 mM calcium chloride dihydrate (CaCl_2_·2H_2_O), all purchased from Sigma. Stock solutions for zebrafish embryos experiments such as PTU, egg water, phosphate buffer saline (PBS), and methylene blue solution were prepared as described in [30,31]. Water was purified using a MilliQ water purification system (Millipore, Guyancourt, France).

### 2.2. Preparation of Photocatalytic Nanocomposites

The photocatalytic compounds were synthesized as described in the Al-Kandari method [12]. The H_2_RGOTi composite was prepared by reducing the TiO_2_-supported GO (GOTi) in a quartz reactor using a flow of H_2_ gas at a rate of 100 mL min^−1^ for 30 min and a temperature of 450 °C [30]. On the other hand, RGOTi, TiO_2_-supported thermally reduced GO, was prepared in a Teflon-lined stainless-steel autoclave. The TiO_2_was added to a mixture of absolute ethanol and sonicated for 30 min. Then, the GO suspension was added under vigorous stirring, and the pH was adjusted to 3.5 using ammonia solution and nitric acid. The suspension was transferred to a Teflon-lined stainless-steel autoclave that was operated at a 120 °C for 24 h. Finally, the suspension was centrifuged, washed with 1 M HCl and deionized water and dried at 80 °C for 24 h as described in [12].

Stock solutions 2.0 g/L for the two photocatalytic nanocomposites (0.1 RGOTi and 0.1 H_2_RGOTi) were prepared by adding 0.02 mg of each compound to 10 mL 1× PBS. Then, all the stock solutions were probe sonicated for 5 min. For the toxicity experiment, the stock solutions were then further diluted in zebrafish PTU-E3 media to the desired tested concentration (100, 200, 300, 400, 600, 800 and 1000 mg/L).

### 2.3. Zebrafish Embryos’ Culture

In our study, we used wild type zebrafish embryos (AB strain). The AB zebrafish were originally purchased in 2014 from the Model Fish Facility (MFU), Norwegian University of Life Sciences, Department of Production Animal Clinical Sciences, Oslo, Norway. The AB wild type strain of zebrafish was maintained in an environmentally controlled lab (Photoperiod: 14 h light/10 h dark cycle with a water temperature of 28 °C) [32] in the zebrafish laboratory at the Biomedical Research Center (BRC), Qatar University, Doha, Qatar. Before spawning, two pairs of male and female fish were placed in a single mating box separated by a divider. Spawning was triggered by removing the divider in the morning of the next day, and the embryos were collected 2 h afterward. Before conducting our experiments, healthy and fertilized embryos were selected and placed in a new Petri dish, and the unhealthy embryos were discarded. All experiments were performed according to the local and international regulations and complied with animal protocol guidelines required by the Qatar University in laboratory animal and Policy on Zebrafish Research that was established by the Department of Research in the Ministry of Public Health, Doha, Qatar.

### 2.4. Acute Toxicity (Acutoxicity) Assays

Stock solutions of 2.0 g/L were prepared for each of the two photocatalytic nanocomposites. Then the stock solutions were sonicated for 5 min to achieve homogenous suspension. Afterward, the two suspensions were further diluted in PTU medium to obtain the desired concentrations (100, 200, 300, 400, 600, 800, 1000 mg/L) as described previously [30]. Basically, at 24-h post fertilization (hpf), embryos were moved to a small Petri dish for dechorionation. The volume of PTU was reduced to cover the surface of the embryos, and 450 µL of 1.0 mg/mL of pronase (Sigma, Steinheim, Germany) was added to the embryos. Then, the embryos were incubated until the chorion started to become soft (~10 min) [33]. After that, embryos were washed three times, and healthy dechorionated embryos were chosen. Each exposure experiment used 12-well plates, and each well composed 3 mL of PTU media containing (i) eight different concentrations (100, 200, 300, 400, 600, 800, 1000 mg/L) of 0.1 RGOTi; (ii) eight different concentrations (100, 200, 300, 400, 600, 800, 1000 mg/L) of 0.1 H_2_RGOTi; (iii) positive control (PC) ZnO (10, 20 mg/L); (iv) Negative Control (NC) PTU. The survival rate and morphological deformities were observed and recorded at 3 time point intervals (48, 72, and 96-hpf) using a standard dissecting microscope. Teratogenicity was scored by gross microscopic assessment (general evaluation, without using a specific software) of each embryo and by calculating the number of dead and deformed embryos (defects in body size, yolk and heart edema, pigmentation, scoliosis, and movement problem) over the number of total embryos used for each concentration. The embryos were considered dead if they demonstrated coagulation of fertilized eggs, lack of somite formation, lack of detachment of tail-bud from the yolk sac and lack heartbeat. The median lethal dose (LC_50_) was calculated by fitting a sigmoidal curve to mortality data at 95% confidence interval using the GraphPad Prism 7 software (version 7.01, San Diego, CA, USA) as described elsewhere [30,31,34,35]. In addition, the NOEC value was designated as the highest tested concentration that had no statistically significant effect within the exposure period when compared with the control (<20% mortality). The LOEC, which is the concentration with mortality or body deformities (embryos with movement difficulties, Scoliosis, Pigmentation, increase/ decrease pericardial size, and yolk size) was greater than or equal to 20% in the embryos treated, were calculated. Twenty-five embryos were used for each tested dose condition of all the nanocomposites and controls. The experiments were repeated 2 independent times.

### 2.5. Zebrafish Embryo Imaging

Zebrafish embryos were imaged using a Zeiss SteREO Discovery V8 Microscope equipped with Hamamatsu Orca Flash high-speed camera and a workstation equipped with HCImage software, version 4.4.1.0 (Hamamatsu Photonics, Tsukuba, Japan). This camera can record image sequences with 100 frames per second (fps) speed. At 72-hpf, desired embryos were removed from a six-well plate and placed on a depression slide of egg water with a small drop of 3% methylcellulose. Embryos were arranged using a probe and hair loop tool; embryos are oriented depending on the region to be analyzed [36]. For measurement of the dorsal aorta (DA) and posterior cardinal vein (PCV), the major blood vessels in a zebrafish, embryos were oriented on their sides. All embryos were imaged at the same magnification and in the same orientation; the head is pointing to the left, heart and yolk sac facing upwards, and tail pointing to the right. A capture/image of the whole embryo was taken for all concentrations of the nanocomposites, including the negative and positive control (NC and PC, respectively) groups. Then, videos were taken off the tail of the embryos at the same site from all groups; ensuring that the two blood vessels were clearly visible. The blood flow in two blood vessels was measured, and then the heart rate was calculated from the results obtained. Variations in the sizes of the yolk, heart and the body length were measured for all the embryos using the ImageJ software version 1.52a (NIH, Washington DC, USA) bundled with Java 1.8.0_172 [30,37].

### 2.6. Cardiotoxicity Assay

Blood flow in the two major blood vessels in the zebrafish embryo, dorsal aorta and PCV, vessels can be imaged easily in the trunk. By tracking RBC movements, it was possible to measure the heartbeat, as well as the average and peak flow velocities in these vessels. RBC tracking can be done by image analysis algorithms.In these experiments, we utilize MicroZebraLab blood flow from Viewpoint (version 3.4.4, Lyon, France). Functional and structural assessment of the cardiovascular system from recorded time-lapse image sequences can be performed by analyzing blood flow in major blood vessels or analyzing the dynamics of the heart movement.

### 2.7. Locomotion (Neuromuscular Toxicity) Assay

In order to evaluate the toxicity of the photocatalytic nanocomposites on the embryos’ neuromuscular system, we assessed the tail flicking activity as previously performed [30]. At 3-hpf, healthy embryos were chosen (25 embryos per well) and moved to 6-well plates and incubated for 24 h in fresh PTU-E3 media containing (i) eight different concentrations (100, 200, 300, 400, 600, 800, 1000 mg/L) of 0.1 RGOTi (ii) eight different concentrations (100, 200, 300, 400, 600, 800, 1000 mg/L) of 0.1 H_2_RGOTi (iii) PC ZnO (10, 20 mg/L). Spontaneous tail coiling was evaluated in embryos aged 24 to 26-hpf. Videos were taken at 24-hpf for the chorions without moving them out of the Petri dish under the ZEISS Stereo Lumar.V12 microscope (Oberkochen, Germany) for 1 min (32 frames per second). Locomotion was evaluated by analyzing the videos using software called DanioScope (version 1.1, Noldus, Wageningen, The Netherlands) as described elsewhere by [30]. By following the protocol of the software, each embryo was marked by a separate arena around it, to detect the movement of the tail inside the chorion optimally. The chorion activity was measured for 10–20 embryos, and the results were compared to the NC and PC.

### 2.8. Hatching Rate Assay

After taking the videos for the embryo locomotion activity within the chorion, the 6-well plates (25 embryos per well) containing the embryos was returned to the incubator. The hatching rate was evaluated at 48 and 72-hpf hours (every 24 h). The hatching rate was calculated as a ratio of the number of hatched embryos divided by total number of incubated embryos ×100 in each well.

### 2.9. Haemoglobin Staining

In order to evaluate the toxic effect of the two photocatalytic compounds on the haemoglobin synthesis process, we performed the o-dianisidine staining (Sigma, Steinheim, Germany) on the embryos as previously described [38]. At 24- hpf, 12 Healthy embryos were dechorionated and incubated for 48 h in fresh PTU-E3 media containing (i) 400, 600 mg/L of 0.1 RGOTi (ii) 400, 600 mg/L of 0.1 H_2_RGOTi (iii) PTU only as NC. At 72-hpf, embryos were washed by PTU-E3 media and stained in the dark for 30 min in o-dianisidine solution containing (0.6 mg/mL o-dianisidine, 40% ethanol, 10 mM sodium acetate (pH 4.5), and 0.65% hydrogen peroxide), fixed in 4% paraformaldehyde in PBS overnight at 4 °C. Embryos were horizontally aligned, positioned on the side and embedded into 3.0% (*w/v*) methyl-cellulose for imaging. Images were taken using Zeiss Axiocam ERc 5s camera under bright field microscopy (Stemi 508 Zeiss, Oberkochen, Germany) at 50×. The intensity and the size of red coloured areas (o-dianisidine stained areas) in the yolk sac of each embryo were quantitated using the ImageJ software version 1.52a.

### 2.10. Statistical Analysis

The cumulative mortality was expressed as a percentage of dead embryos for 96-hpf. Descriptive statistics (DS) such as mean (*m*) and standard deviation (SD) were calculated for the locomotion assay, hatching rate, o-dianisidine, and cardiotoxicity assay, data were presented as mean ± SD. Statistical analysis was performed with the one-way analysis of variance (ANOVA) followed by the Dunnet test as compared to NC. The Chi-square test was used to calculate the significance between the percentages compared to NC for the hatching rate. All significant outliers were removed by GraphPad Prism 7 software. Finally, values outside of the interval: 2* Standard Deviation < *X* < 2* Standard Deviation, where *X* is the measured value, were considered as outliers and removed from statistical analysis. Significance (*) = *p* < 0.05; (**) = *p* < 0.01; (***) = *p* < 0.001.

## 3. Results and Discussion

### 3.1. General Acutoxicity Assessment (Median Lethal Concentration (LC_50_), No Observed Effect Concentration (NOEC), and Lowest Observed Effect Concentration (LOEC))

**Figure 1:**
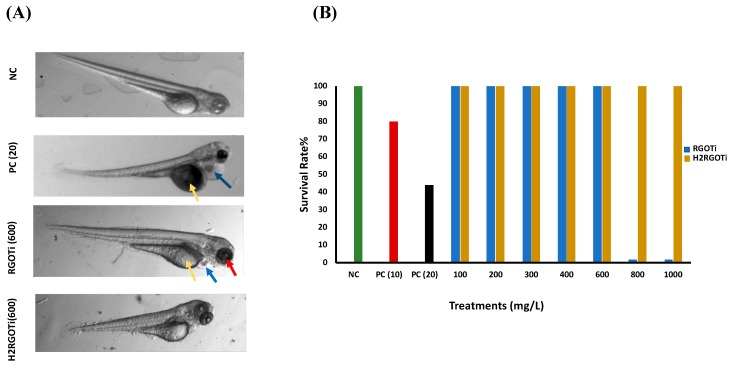
(**A**) Representative pictures (96-hpf) of acute toxicity experiments of ZnO nanoparticles-exposed embryos (positive control, PC), N-phenylthiourea (PTU) (negative control, NC) and the photolytic nanocomposites. Note the deformed embryos in 20.0 mg/L of the PC (yolk (yellow arrow) and cardiac edema (blue arrow)), thermally-reduced graphene oxide/TiO_2_ semiconductor photocatalyst (RGOTi) (yolk, cardiac and increased eye size (red arrow)) and hydrogen-reduced graphene oxide/TiO_2_ semiconductor photocatalyst (H_2_RGOTi) (smaller body size). (**B**) Acute toxicity and survival rate of embryos exposed at different concentrations of RGOT and H_2_RGOTi nanocomposites compared to the PC and NC, n = 50. Note: the edema in the hart and yolk and the eye size was measured by the ImageJ software. All the images were taken at the same magnification (20×).

We first examined the potential adverse effect of the two photocatalytic nanocomposites on the zebrafish embryos gross development using an acute toxicity assay adapted by the Organization of Economic Co-operation and Development [39] guidelines for testing chemical toxicity (N° 203 and 236). Previous studies have shown that the early developmental stages of zebrafish are the most sensitive to external nanocomposites or drugs [40,41,42,43,44]. Therefore, we chose the embryonic period from 24 to 96-hpf as the administration time to study the potential toxicity of these photocatalytic nanocomposites. The percentage of cumulative survival was measured at 96-hpf, which is the recommended observation time [45]. The NOEC (i.e., mortality/teratogenicity score <20%) for ZnO was less than 10 mg/L, while the LOEC (mortality or teratogenicity score >20%) was at 10 mg/L, showing a survival rate of 78% (Figure 1B). The calculated LC_50_ value for the ZnO was 13.29 mg/L. RGOTi, significant mortality could be observed starting at 800 mg/L and above that concentration (Figure 1A,B). The calculated LC_50_ for RGOTi was 748.6 mg/L. No significant mortality/teratogenicity could be detected at concentrations below 400 mg/L. Thus the NOEC was assumed to be less than 400 mg/L. However, starting from 400 mg/L, and for all concentrations above 400 mg/L, the mortality/teratogenicity was greater than 20%, thus the LOEC for RGOTi was assumed to be at 400 mg/L (Figure 1A and Figure S1). For H_2_RGOTi, no significant mortality was observed at all tested concentrations (Figure 1B). Thus, the calculated hypothetical LC_50_ for H_2_RGOT is estimated to be above 1000 mg/L, the highest tested concentration. Although the NOEC and the LOEC for H_2_RGOTi were similar to RGOTi, H_2_RGOTi showed less severe teratogenic phenotypes than the RGOTi (Figure 1A and Figure S1). In conclusion, according to the Fish and Wildlife Service Acute Toxicity Rating Scale (Table S1), RGOTi should be classified as “practically not toxic” and H_2_RGOTi as “relatively harmless”.

Based on our acute toxicity results, a concentration of 400 and 600 mg/L was selected for the 2 photocatalytic compounds in the consequent experiments to test their specific toxicity on zebrafish embryos.

### 3.2. Quantitative Assessment of Specific Teratogenic Phenotype Exerted by Thermally-Reduced Graphene Oxide/TiO_2_ Semiconductor Photocatalyst (RGOTi) and Hydrogen-Reduced Graphene Oxide/TiO_2_ Semiconductor Photocatalyst (H_2_RGOTi)

**Figure 2:**
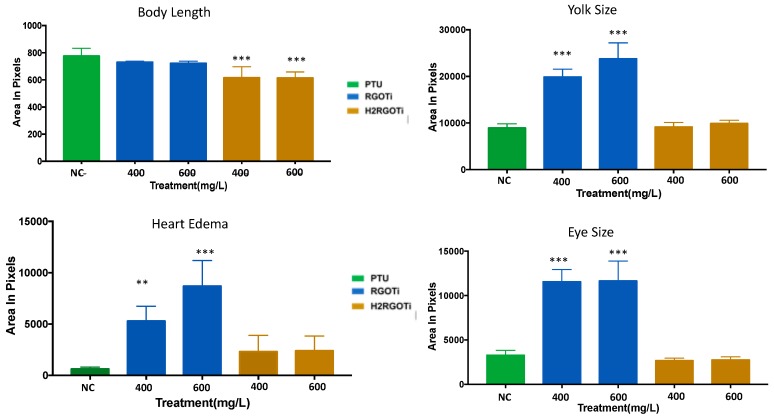
Specific teratogenic changes induced by RGOTi and H_2_RGOTi. Average body length, yolk, heart, and eye size were measured using ImageJ software version 1.52a. Twenty-five embryos were used per concentration. One-way analysis of variance (ANOVA) was used to compare the differences between the average of the imaged areas between groups. ** *p* < 0.01 and *** *p* < 0.001, *n* = 10.

Although the two nanocomposites did cause significant mortality only at high concentration (800 mg/L for RGOTi and higher than 1000 mg/L for H_2_RGOTi), we wanted to investigate whether any other potentially harmful effects other than mortality could be elicited at lower concentrations. For this purpose, for any individual embryo (i) standard body length, (ii) eye size (ocular area), (iii) pericardial area, and (iv) yolk size were determined from images captured by HCImage software, version 4.4.1.0 (Hamamatsu Photonics, Tsukuba, Japan) and analyzed with the ImageJ software version 1.52a. For body length (i) embryos treated with 400 and 600 mg/L H_2_RGOTi, but not with RGOTi, showed a significant decrease in the body length (shortened tail stem length) as compared to NC (Figure 2). This suggest that H_2_RGOTi might have induced developmental defects in the neural structures such as the spine, which could be associated with early defects in somite formation ultimately leading to deformities of the musculature and skeleton [46], which is in consonance with the finding that most of the H_2_RGOTi-treated embryos showed decreased movement activities at 96-hpf (Figure S1). Moving to the eye size (ii), no significant difference was detected at 400 and 600 mg/L for H_2_RGOTi (Figure 2). However, the RGOTi-treated embryos showed a significant increase in eye size at 400 and 600 mg/L compared to NC. The pericardial area (iii) is a measure of fluid accumulation (edema) in the pericardial space, as an indicator of heart failure and other forms of cardiac dysregulation [47]. While embryos exposure to H_2_RGOTi failed to significantly affect pericardial size as compared to NC, RGOTi-treated embryos showed a significant dose-dependent increase in pericardial size at both 400 and 600 mg/L, suggesting a specific cardiotoxic effect of RGOTi. As for what concerns the yolk size (iv), embryos exposition to RGOTi, but not to H_2_RGOTi, elicited a significant increase in the yolk sac size at both 400 and 600 mg/L, suggesting that RGOTi may impact the normal metabolism and nutrient intake of the treated embryos, ultimately leading to nutrient accumulation and fluid retention in the yolk sac [48,49]. In conclusion, similar to our findings in “Section 3.1”, these new findings provide another line of evidence that H_2_RGOTi could be more ecofriendly than RGOTi, which showed more severe specific teratogenic phenotypes (increased eye size, pericardial and yolk edema) than H_2_RGOTi, which only induces changes in the body size (Figure 1A and Figure S1).

### 3.3. Assessment of RGOTi and H_2_RGOTi Potential Cardiotoxicity by Heart Rate Quantitation

**Figure 3:**
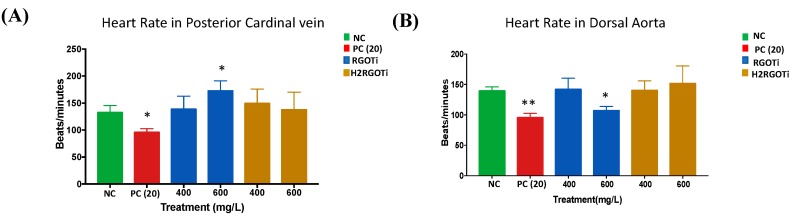
Effect of RGOTi and H_2_RGOTi on heart rate. Heart rate was calculated from the PCV (**A**) and dorsal aorta (**B**) of the embryos following treatment with each indicated concentration. Nineteen embryos were used per concentration. One-way ANOVA was used to compare the differences between the average of the imaged areas between groups. * *p* < 0.05 and ** *p* < 0.01, *n* = 10.

Zebrafish has been reported to be an excellent model to study cardiotoxicity and other heart dysfunctions induced by drugs and other compounds [49,50]. The dorsal aorta is the major trunk axial artery and is one of the first vessels to assemble during early development in all vertebrates. The dorsal aorta forms immediately below the notochord and above the posterior cardinal vein (PCV), which is also known as the major trunk axial vein in Zebrafish. Our findings showed that H_2_RGOTi did not affect cardiac development as highlighted by normal PCV heart rate and dorsal aorta morphology (Figure 3). While there was a significant difference in the heart rate for 600 mg/L RGOTi-treated embryos in both vessels, PCV and dorsal aorta (Figure 3). This rate difference was not surprising, because our previous results in Section 3.2 showed that RGOTi-treated embryos also cause yolk edema, which may lead to heart rate dysfunction.

### 3.4. Assessment of RGOTi and H_2_RGOTi Potential Toxicity on Neuromuscular Activity by Locomotion (Tail Coiling) Assay

**Figure 4:**
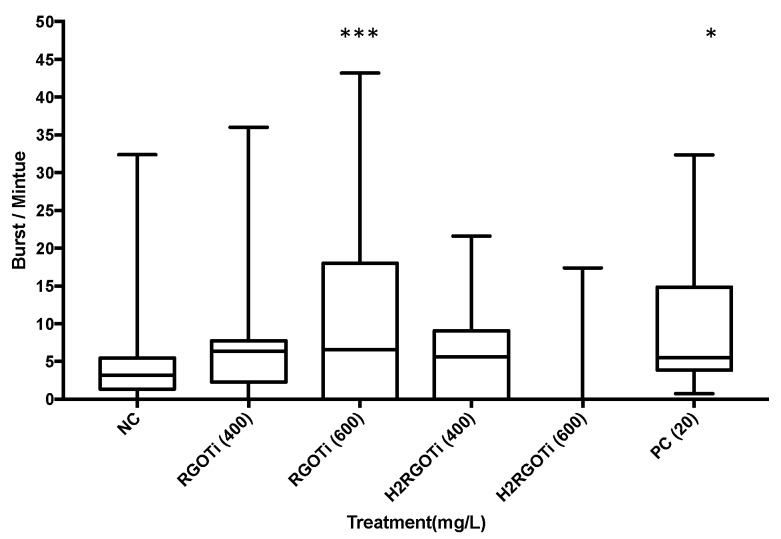
Assessment of potential neuro/muscular toxicity at 24-hpf by locomotion/tail coiling assay. The plot represents the average tail coiling (burst/min) measured by DanioScope software. Twenty-five embryos were used per each concentration. One-way ANOVA was used to compare the differences between groups. *n* = 25. (*) = *p* < 0.05; (***) = *p* < 0.001.

The activity and the locomotion of the embryos within the chorion at 24-hpf is an indirect indication of the musculature or nervous system development and can be used to investigate the effect of drugs or other compounds on these systems [30,51,52]. Spontaneous tail coiling starts from 17-hpf and peaks at 19-hpf, followed by a phase of response to touch at 21-hpf. A normal zebrafish embryo at 24-hpf shows 3–5 spontaneous burst/min followed by a period of inactivity [52] Here, the spontaneous tail coiling of each embryo was measured using the DanioScope software [30,53]. A dose-dependent increase in spontaneous tail coiling was observed in RGOTi-treated embryos (Figure 4). As shown in Figure 4, 600 mg/L of RGOTi elicited a significant increase in the tail coiling activity (9.8 burst/min) as compared to NC, which is normally between 3–5 burst/min [54]. On the other hand, H_2_RGOTi did not evoke any significant effect on the tail coiling activity as compared to NC. Studies have shown that an increase in the number of the spontaneous tail coil at 24-hpf is an indicator of the embryo’s hyperactivity and twitching, which are considered epileptic movements [55,56,57]. On the other hand, the inhibition of tail coiling motility is an indication of a structural or functional defect in musculature development [58]. Thus, the increased tail coiling activity elicited by RGOTi (but not H_2_RGOTi) suggests that, rather than musculature development, this photocatalytic compound might adversely affect the nervous system activity of the embryos.

### 3.5. Assessment of RGOTi and H_2_RGOTi Potential Toxicity on Hematopoietic Activity Using O-Dianisidine Staining

**Figure 5:**
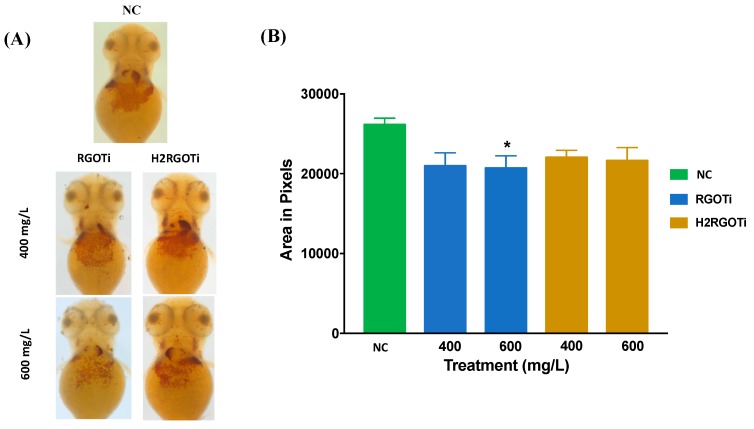
Distribution of haemoglobin- positive cells was detected by O-dianisidine staining at 72-hpf. (**A**) Representative images of O-dianisidine Stain concentrated on the yolk sac of the NC, RGOTi (400, 600 mg/L), and H_2_RGOTi (400, 600 mg/L). The images were captured using bright field microscopy, at 50×. (**B**) Quantification analysis of the erythrocytes number in the areas stained by o-dianisidine. Embryos treated with RGOTi, 600 mg/L showed a significant decrease in the hematopoietic activity as compared to NC. One-way ANOVA was used followed by the Dunnet test to compare the difference between the treated groups. *n* = 20, * *p* < 0.05

The peroxidase activity of the haemoglobin in the erythrocytes could be used as an indicator of hematopoiesis [38]. The o-dianisidine stain is used as a direct measurement of haemoglobin synthesis and could be used as well for indirect measurement of erythrocytes synthesized by the bone marrow (erythropoiesis) [38]. Only positive haemoglobin cells will take up the stain. The higher the hematopoietic activity, the larger the area measured [38]. Embryos exposure to 600 mg/L of RGOTi induced a significant decrease in haemoglobin syntheses by red blood cells or due to a decreased number of red blood cells generated by the bone marrow (Figure 5). Very few studies used this assay in evaluating the potential toxicity of chemicals. A study done by Leet [59] and his colleagues showed that the protoporphyrinogen oxidase inhibitor butafenacil completely eliminated haemoglobin production and circulation. The reason behind that could be due to the blockage in heme biosynthesis and, as a result, a reduction of haemoglobin and erythrocytes production within zebrafish embryos. Similarly, butafenacil treatment in rodent models (rats and mice) resulted in decreased haemoglobin, hematocrit, and mean corpuscular volume [60].

### 3.6. Hatching Rate

**Figure 6:**
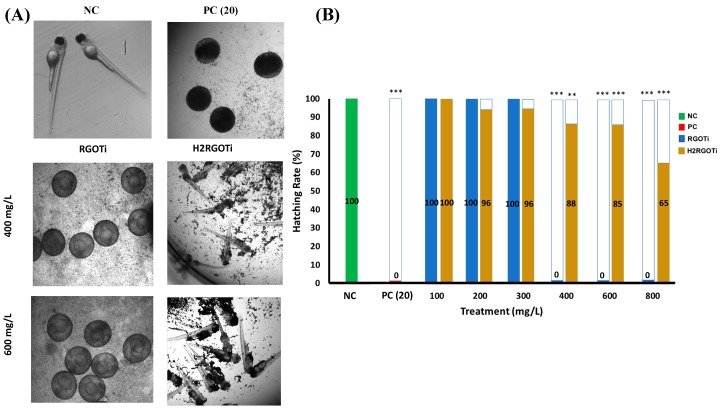
The effect of photocatalytic compounds on the zebrafish embryos hatching rate. (**A**) Representative pictures for the hatching rate at 96-hpf. 400 and 600 mg/L of RGOTi showed complete inhibition of the zebrafish embryos hatching rate by 96-hpf. However, embryos treated with H_2_RGOTi eventually hatch by 96-hpf. (**B**) Embryos treated with H_2_RGOTi showed a dose-dependent decrease in the hatching rate. The Chi-square test was used to calculate the significance between the percentages compared to NC, *n* = 50. (*) = *p* < 0.05; (**) = *p* < 0.01; (***) = *p* < 0.001.

The hatching rate is a critical indicator for estimating the developmental state of the zebrafish embryo [32]. The normal hatching period for zebrafish embryos is between 48 to 96-hpf. Embryos treated with RGOTi showed a drastic inhibition in the hatching rate at 400 mg/L onwards (Figure 6). However, H_2_RGOTi-treated embryos showed a more gradual and less severe delay in hatching following treatment. Although the specific mechanism for the delay of hatching is not yet known, the observed delay of the hatching rate after embryo treatment could be due to several reasons. First, the hardening of the chorion and consequently the lower permeability to the chemicals may act as a protective mechanism towards the photocatalytic compounds [61,62]. Second, it is notable that similar delays in the hatching of the embryos usually occur when the neurotransmitter levels are affected [63]. Third, both composites could affect the enzymatic action of *chorionase*, which is a critical protease involved in the control of embryos hatching [64]. Fourth, it could be due to the fact that both nanocomposites weakened the muscular system of the embryos and this may have also contributed to the failure in hatching as the mechanical force of the embryos’ movement on the walls of the chorion normally causes rupture. The findings of the hatching rate experiment are consistent with all of the above experiments, where we showed that RGOTi has a more drastic effect on embryo development than H_2_RGOTi.

RGOTi at 400 mg/L caused a significant delay in the hatching rate, but without causing significant mortality. Maybe this is due to the following reasons: (i) although RGOTi does not cause significant mortality at 400 mg/L, it induced significant teratogenicity at a concentration higher than 400 mg/L, which might affect hatching rate; (ii) the hatching rate experiment was done differently from the mortality experiment. In the mortality experiment, the embryos were treated at 24-hpf after the chorion was removed by the pronase enzyme. However, in the hatching rate experiment, the treatment was started at 4-hpf without dechorionation. Thus, the chorion maybe was acting as a protective shield that prevents exposure of these embryos to the nanoparticles; and (iii) the mortality was scored at 96-hpf, and although these embryos were not able to hatch at 72-hpf, most of them were able to hatch at later time (96-hpf), or even stayed alive within the chorion without being hatched. We were not sure what is going to happen to the embryos after 96-hpf, which is the end-point of all of our experiments. Summarizing our experiments, we conclude that although these nanoparticles delayed the hatching rate, maybe by hardening the chorion, they are unable to cause significant mortality at 96-hpf. 

The tested concentrations do have environmental relevance. Indeed, they have been chosen in order to be within the Fish and Wildlife Service Acute Toxicity Rating Scale [65], that classified a compound’s toxicity according to LC_50_ as follows: highly toxic from 0.1–1.0 mg/L, 1.0–10 mg/L moderately toxic, 10–100 mg/L slightly toxic, 100–1000 mg/L practically nontoxic, and >1000 mg/L is relatively harmless. In our case, it is quite hard to know the actual concentration of these nanoparticles, if leakage into potable water (lakes, rivers, springs, etc.) happen. Although it is reasonable to assume that in this situation the compounds could be more diluted as compared to tested concentrations, we cannot exclude that in the case of particular occurrences (i.e., pipeline ruptures or leakage) the compounds’ concentration in the microenvironment nearby the pipeline may reach values even higher than those we tested in this study. Generally, RGOTi showed a more toxic chemical profile than did H_2_RGOTi, as the severity of the embryos’ phenotype was more profound. Due to the compounds being of identical chemical structures and make-up, it can, therefore, be concluded that the method of reduction of the compounds was what differentiated their impacts. The leftover traces of chemicals on the surface of RGOTi is mainly responsible for its high toxicity compared to the H_2_RGOTi, which was cleanly reduced using a flow of hydrogen gas.

## 4. Conclusions

The use of an effective and economical removal technique for phenolic compounds in wastewater is an urgent demand. Photocatalysis using TiO_2_-based nanocomposites is one of the advanced oxidation processes that proved to be highly efficient in the deterioration of a diversity of refractory pollutants into decomposable compounds, which are finally mineralized to water and carbon dioxide [9,12]. To our knowledge, the present work is the first to report a full toxicity evaluation of a reduced graphene oxide/TiO_2_ nanocomposite photocatalyst prepared by two different methods RGOTi (hydrothermally reduced graphene oxide) and H_2_RGOTi (reduction of graphene oxide with H_2_ gas at 450 °C) on a freshwater animal model. The toxicity tests performed indicated that RGOTi is much more toxic than H_2_RGOTi as it induced severe toxicity in all the parameters tested. In this context, embryos treated with RGOTi showed a drastic inhibition in the hatching rate at 600 mg/L onward. However, H_2_RGOTi treated embryos showed a more gradual and less severe delay in hatching following treatment. Dose-dependent increase in spontaneous tail coiling was observed with RGOTi-treated embryos at 24-hpf. On the other hand, H_2_RGOTi did not evoke any significant effect on the tail-coiling activity as compared to NC. Consistent with the above results are the findings revealing that there was a significant difference in the heart rate for embryos treated with 600 mg/L of RGOTi in both vessels DA and PCV. While H_2_RGOTi did not show any significant influence at any concentration on heart rate variability measures. Finally, the O-dianisidine stain demonstrated that treatment with 600 mg/L of RGOTi showed a significant decrease in hematopoietic activity in the embryos, which could be a result of the toxicity of the compound that may have interfered with this process. From our study, we can conclude that the reduction method during the preparation of the photocatalytic-reduced graphene oxide/TiO_2_ nanocomposite could play a fundamental role in their toxicity on aquatic life.

## Supplementary Materials

The following are available online at https://www.mdpi.com/2079-4991/9/4/488/s1, Table S1: Acute Toxicity Rating Scale by Fish and Wildlife Service (FWS), Figure S1: Graphical representation of the teratogenic effects observed in the embryos treated with RGOTi and H_2_RGOTi.
