# Supplementary material for: Ecotoxicological Assessment of Thermally- and Hydrogen-Reduced Graphene Oxide/TiO2 Photocatalytic Nanocomposites Using the Zebrafish Embryo Model
Source: Nanomaterials (Basel). 2019 Mar 28;9(4):488. doi: 10.3390/nano9040488 (PMC6523634; doi:10.3390/nano9040488)
Supplement: Supplementary file 1 [file nanomaterials-09-00488-s001.pdf]

Table S1. Acute Toxicity Rating Scale by Fish and Wildlife Service (FWS) [1].

Relative Toxicity	Aquatic LC_{50} (mg/L)
Super Toxic	0.01–0.1
Highly Toxic	0.1–1
Moderately Toxic	1–10
Slightly Toxic	10–100
Practically Nontoxic	100–1000
Relatively Harmless	>1000

General Teratogenic Effect Assessment by Gross microscopic examination

Embryos treated with 100 and 200 mg/L of RGOTi did not show any teratogenic effects. At 400 mg/L and above all of the embryos had pericardial edema and movement difficulties. The scoliosis teratogenic effect started at 400 mg/L (80% of the embryo) and increased in dose dependent manner. The yolk edema teratogenic effect was evident at 600 mg/L and increased at doses dependent manner, suggesting specific effect of the RGOTi. Embryos exposure at 100 and 200 mg/L of H_2RGOTi also failed to induce teratogenic effects. H_2RGOTi-elicited teratogenic effect was less severe than RGOTi, as scoliosis and body deformities (other than heart edema) was absent at dose 400 and 600 mg/L. The yolk edema teratogenic effect was started at 800 mg/L for both compounds. These results suggests that up 200 mg/mL, these nanocomposite has no adverse effect on embryonic development of zebrafish embryos.

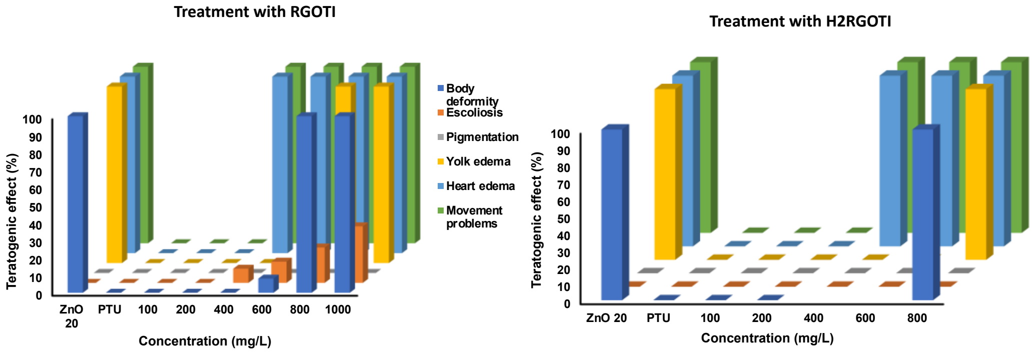

Figure S1. Graphical representation of the teratogenic effects observed in the embryos treated with RGOTi and H_2RGOTi.

References

1 El-Harbawi, M. Toxicity Measurement of Imidazolium Ionic Liquids Using Acute Toxicity Test. *Procedia Chem.* **2014**, *9*, 40–52.
